# Challenges in Differential Diagnosis and Management of Lymphoepithelial Sialadenitis (LESA): A Scoping Review

**DOI:** 10.3390/life16071199

**Published:** 2026-07-20

**Authors:** Miruna Bratiloveanu, Mihai Dumitru, Bogdan Banica, Oana Maria Patrascu, Crenguta Serboiu, Andreea Marinescu, Alina Oancea, Daniela Vrinceanu, Adrian Costache

**Affiliations:** 1 Pathology Department, Carol Davila University of Medicine and Pharmacy, 020021 Bucharest, Romania; mirunabratiloveanu@gmail.com (M.B.); adriancostacheeco@yahoo.com (A.C.); 2 ENT Department, Carol Davila University of Medicine and Pharmacy, 020021 Bucharest, Romania; vrinceanudana@yahoo.com; 3 OMF Department, Bucharest University Emergency Hospital, 050098 Bucharest, Romania; bogdan.ddr@gmail.com; 4 Histology and Molecular Biology Department, Carol Davila University of Medicine and Pharmacy, 020021 Bucharest, Romania; crengutas@yahoo.com; 5 Radiology and Diagnostic Imaging Department, Carol Davila University of Medicine and Pharmacy, 020021 Bucharest, Romania; andreea_marinescu2003@yahoo.com; 6 ENT Department, Elias Clinical Hospital, 011461 Bucharest, Romania; dr.alina.oancea@gmail.com

**Keywords:** lymphoepithelial, sialadenitis, LESA, salivary, gland

## Abstract

**Background:** Lymphoepithelial sialadenitis (LESA) is a chronic lymphoid-rich inflammatory disorder of the salivary glands that is strongly associated with Sjögren’s disease and may overlap clinically, radiologically, and histopathologically with IgG4-related sialadenitis, HIV-associated lymphoepithelial lesions, chronic sialadenitis, salivary gland tumors, and extranodal marginal zone B-cell lymphoma of mucosa-associated lymphoid tissue (MALT lymphoma). **Objective:** This scoping review mapped current evidence on diagnostic challenges, differential diagnostic criteria, management strategies, and surveillance considerations for LESA. **Eligibility criteria:** Sources were selected using the Population–Concept–Context framework and included the literature addressing salivary gland lymphoepithelial lesions, LESA, Sjögren’s disease-associated salivary gland involvement, or related lymphoid-rich salivary gland disorders published from 2010 onward. **Sources of evidence and charting methods:** Google Scholar was searched, records were screened in sequential stages, and relevant data were charted narratively across clinical, serological, imaging, histopathological, immunophenotypic, molecular, therapeutic, and follow-up domains. **Results:** Thirty-seven sources were included. The evidence indicates that LESA is usually characterized by chronic lymphoplasmacytic inflammation, acinar atrophy, lymphoepithelial lesions, and preserved lobular architecture; however, these findings may overlap with early or established MALT lymphoma. Immunohistochemistry, assessment of light-chain restriction, clonality testing, serological markers, and imaging are useful adjuncts, but no single test is independently definitive. Conservative management and symptomatic care are appropriate for stable disease, whereas corticosteroids, immunomodulatory therapy, sialendoscopy, surgery, radiotherapy, or systemic lymphoma therapy may be considered according to clinical context. **Conclusions:** LESA requires integrated clinicopathological interpretation and multidisciplinary follow-up. Key evidence gaps include the absence of standardized LESA-specific diagnostic criteria, limited validation of molecular and flow cytometric approaches in salivary gland specimens, and lack of consensus surveillance protocols.

## 1. Introduction

Lymphoepithelial sialadenitis (LESA) represents a diagnostically challenging lymphoid-rich disorder of the salivary glands, most often involving the parotid gland. Although classically defined by dense lymphoid infiltration with epithelial involvement, its clinical relevance extends beyond histological recognition because it frequently overlaps with Sjögren’s syndrome (SS), IgG4-related sialadenitis, HIV-associated parotid disease, chronic sialadenitis, benign lymphoepithelial cysts, salivary gland tumors with lymphoid stroma, and extranodal marginal zone B-cell lymphoma of mucosa-associated lymphoid tissue (MALT lymphoma). Consequently, the introduction of this review is directed toward the central diagnostic dilemma: how to distinguish benign autoimmune or inflammatory lymphoepithelial lesions from malignant or premalignant lymphoid proliferations in daily clinical practice [1].

This dilemma is clinically important because LESA does not present with a single pathognomonic feature. Patients may show recurrent or persistent salivary gland swelling, sicca symptoms, autoimmune serology, cystic or solid imaging findings, and lymphoid-rich biopsy specimens, all of which may also be encountered in several mimicking conditions. Therefore, the diagnostic process requires integration of clinical presentation, imaging, serological markers, histopathology, immunohistochemistry, and, in selected cases, molecular testing [2].

The differential diagnosis is particularly broad in patients with parotid enlargement or lymphoepithelial lesions on biopsy. SS-associated glandular disease may resemble isolated LESA, whereas IgG4-related sialadenitis may show dense lymphoplasmacytic inflammation but is characterized by storiform fibrosis, obliterative phlebitis, and increased IgG4-positive plasma cells. HIV-associated benign lymphoepithelial cysts, chronic obstructive sialadenitis, tumor-associated lymphoid proliferation, and low-grade salivary gland lymphomas may further complicate interpretation, especially when tissue samples are limited [3].

From a clinical standpoint, LESA most commonly presents as recurrent, firm, and usually painless enlargement of the affected salivary gland, which may be unilateral or bilateral. However, this pattern is not specific. Bilateral gland involvement may suggest an autoimmune background, particularly SS, while a persistent, asymmetric, enlarging, or nodular parotid lesion raises concern for lymphoma or another neoplastic process. Sicca symptoms, fatigue, arthralgia, and systemic manifestations should therefore be interpreted in relation to the broader differential diagnosis rather than viewed as diagnostic in isolation [4].

Physical examination and imaging add important but incomplete information. LESA-related lesions may appear as solid, cystic, or heterogeneous parotid abnormalities and may show poorly defined margins or deep gland involvement. Nevertheless, similar findings may occur in inflammatory, autoimmune, cystic, and malignant salivary gland diseases. This overlap explains why radiological assessment must be correlated with serological and tissue-based findings, particularly when differentiating LESA from MALT lymphoma and other lymphoid-rich mimics [5].

Histopathology remains central to diagnosis, but it is also the source of many pitfalls. LESA is characterized by chronic lymphoplasmacytic infiltration, acinar atrophy, preserved lobular architecture, and lymphoepithelial lesions; however, similar lymphoepithelial changes may be seen in the setting of evolving or established MALT lymphoma. Immunohistochemistry, assessment of light-chain restriction, evaluation of B- and T-cell distribution, and molecular clonality studies may help clarify difficult cases, although monoclonality alone does not always equate to overt lymphoma in autoimmune salivary gland disease [6].

The need for accurate differentiation is reinforced by the risk of lymphomatous transformation, particularly to MALT lymphoma. Patients with SS and persistent salivary gland enlargement, cryoglobulinemia, low complement levels, rheumatoid factor positivity, lymphadenopathy, or other systemic risk factors require careful surveillance. Thus, the diagnostic pathway must not only establish whether LESA is present, but also determine whether the lesion is reactive, autoimmune-associated, lymphoma-associated, or already malignant [7].

Histologically, LESA is characterized by a dense, chronic lymphoplasmacytic infiltrate that replaces normal salivary gland parenchyma, leading to acinar atrophy and the formation of lymphoepithelial lesions (LELs). The inflammatory infiltrate is initially multifocal and periductal, progressing to diffuse involvement with the formation of reactive lymphoid follicles and germinal centers. As the disease advances, there is partial or total destruction of acini, with only ductal structures remaining [8].

The hallmark of LESA is the lymphoepithelial lesion, formed by the proliferation of ductal epithelial remnants permeated by lymphocytes. These epithelial nests, or epimyoepithelial islands, consist of hyperplastic ductal cells infiltrated by both T and B lymphocytes, with a predominance of B cells in later stages. Eosinophilic, hyaline basement membrane-like material may be present among the cells of the lesion. Despite extensive inflammation, the overall lobular architecture of the gland is typically preserved, distinguishing LESA from malignant processes that disrupt glandular organization [9].

Given the rarity of LESA, inconsistent terminology, overlapping clinicopathological features, and absence of standardized diagnostic and management pathways, a scoping review is needed to map the available evidence around these diagnostic dilemmas and differential diagnostic boundaries. Accordingly, the objectives of this scoping review are to synthesize current evidence on the clinical, serological, imaging, histopathological, immunophenotypic, and molecular features of LESA; clarify its distinction from SS-related disease, IgG4-related sialadenitis, HIV-associated lesions, chronic sialadenitis, salivary gland tumors, and MALT lymphoma; review available management and surveillance strategies; and identify evidence gaps requiring future research.

## 2. Materials and Methods

This scoping review was designed to map the available evidence on lymphoepithelial sialadenitis (LESA), with particular emphasis on diagnostic challenges, differential diagnosis, management, and surveillance. The review was conducted in accordance with the Joanna Briggs Institute (JBI) methodological guidance for scoping reviews and reported following the Preferred Reporting Items for Systematic Reviews and Meta-Analyses extension for Scoping Reviews (PRISMA-ScR). A scoping review design was selected because the literature on LESA is heterogeneous, uses variable terminology, and spans clinical, radiological, pathological, molecular, and therapeutic domains. Therefore, the objective was to identify and organize the breadth of available evidence rather than to estimate pooled effects or grade certainty of evidence.

The review question was formulated as follows: What evidence is available regarding the diagnostic challenges, differential diagnostic criteria, management strategies, and surveillance considerations for LESA, particularly in distinguishing it from Sjögren’s disease, IgG4-related sialadenitis, HIV-associated lymphoepithelial lesions, chronic sialadenitis, salivary gland tumors, and salivary gland MALT lymphoma?

Eligibility criteria were structured using the Population–Concept–Context (PCC) framework recommended for scoping reviews. The population included patients, case reports, cohorts, reviews, and diagnostic discussions involving salivary gland lymphoepithelial lesions, LESA, Sjögren’s disease-associated salivary gland involvement, or related lymphoid-rich salivary gland disorders. The concept included diagnostic criteria, differential diagnosis, imaging findings, histopathological and immunophenotypic features, molecular findings, management strategies, surveillance, and risk of progression to MALT lymphoma. The context included clinical, radiological, pathological, and multidisciplinary diagnostic settings relevant to major and minor salivary glands. The literature published from 2010 onward was prioritized to reflect contemporary imaging, immunohistochemical, molecular, and clinicopathological practice. Sources were considered eligible when they addressed at least one PCC component relevant to LESA diagnosis, differential diagnosis, management, or follow-up.

The protocol for this scoping review was registered on the Open Science Framework (OSF) under the registration identifier hg7d6. Formal critical appraisal of individual sources was not undertaken, consistent with the purpose of a scoping review, which was to map the extent, range, and nature of the evidence rather than to determine certainty of effect estimates.

Google Scholar was used as the information source for the search. The search strategy combined terms related to “lymphoepithelial sialadenitis,” “LESA,” “salivary gland lymphoepithelial lesion,” “Sjögren,” “MALT lymphoma,” “IgG4-related sialadenitis,” “HIV-associated parotid disease,” “diagnosis,” “histopathology,” “immunohistochemistry,” “molecular,” “management,” and “surveillance.” The most recent search was performed during manuscript preparation. Limits were applied sequentially to retain the literature published from 2010 onward and to prioritize article-type sources relevant to the PCC framework. Records focused on non-salivary or mediastinal lymphoepithelial lesions were excluded because they did not match the review context.

Records retrieved from the search were screened in sequential stages. First, titles and abstracts were reviewed to remove clearly irrelevant sources. Full texts were then assessed against the PCC framework, publication period, article type, and salivary gland relevance. Records were imported into a shared online database. Two reviewer groups (M.D. and B.B.; O.M.P. and C.S.) independently screened titles, abstracts, and full texts, and disagreements were resolved by two additional reviewers (A.M. and A.O.). Data from included sources were charted narratively according to predefined domains: clinical presentation, serology, imaging, biopsy approach, histopathology, immunophenotype, molecular findings, differential diagnosis, management, surveillance, and evidence gaps.

## 3. Results

**Figure 1:**
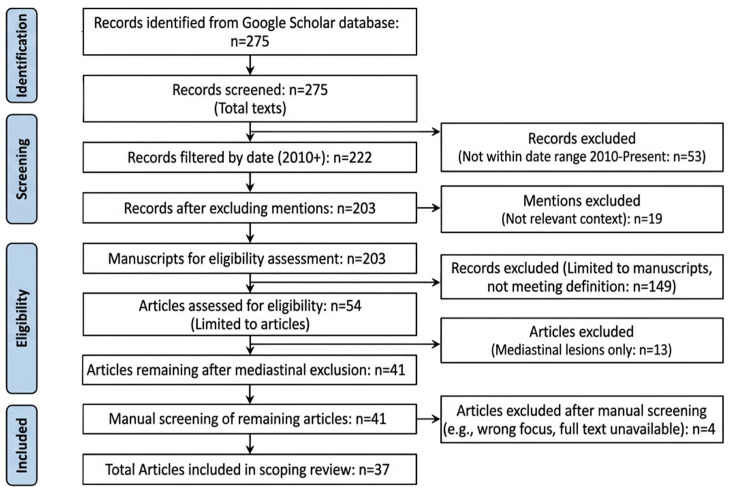
PRISMA flow chart of the present scoping review on LESA.

The search identified 275 records. After applying the 2010 publication-date limit, 222 records remained. Exclusion of records that only mentioned the topic without providing relevant diagnostic, pathological, clinical, or management information left 203 records. Restriction to article-type sources yielded 54 manuscripts. After excluding articles focused on mediastinal or non-salivary lymphoepithelial lesions, 41 articles remained for full-text review. Manual eligibility assessment resulted in 37 sources included in this scoping review. The selection process is summarized in Figure 1. Records were imported into a shared online database. Two reviewer groups (M.D. and B.B.; O.M.P. and C.S.) independently screened titles, abstracts, and full texts. Disagreements were resolved by two additional reviewers (A.M. and A.O.). Data were charted narratively according to predefined domains: clinical presentation, serology, imaging, biopsy approach, histopathology, immunophenotype, molecular findings, differential diagnosis, management, surveillance, and evidence gaps.

### 3.1. Immunophenotype and Molecular Features

Immunohistochemically, the lymphoid infiltrate in LESA is polyclonal, comprising both CD3+ T cells and CD20+ B cells, along with polytypic plasma cells. Germinal centers are surrounded by mantle zone B cells (CD20+, IgM+, IgD+), and interfollicular areas are occupied by T cells. In early disease, T cells predominate, while B cells and plasma cells become more prominent in advanced stages [10].

Molecular studies may reveal clonal immunoglobulin gene rearrangements in up to 40–50% of LESA cases, but this finding does not necessarily indicate overt lymphoma. The presence of monoclonal B-cell populations in the absence of morphological evidence of malignancy is a recognized phenomenon in autoimmune diseases and should be interpreted with caution [11].

### 3.2. Comparison with Minor Salivary Gland Biopsies

In minor (labial) salivary gland biopsies, the histological features of LESA are similar but less pronounced. Lymphoepithelial lesions and germinal centers are less common, and the focus score (number of lymphoid aggregates per 4 mm^2^) is used as a diagnostic criterion for SS. Adequate biopsy samples should include at least 4 mm^2^ of glandular tissue for reliable assessment [12].

### 3.3. LESA Association and Overlap with Sjögren’s Disease

LESA is most frequently encountered in the context of primary SS, an autoimmune disorder characterized by lymphocytic infiltration and destruction of exocrine glands, leading to sicca symptoms. The pathogenesis involves both humoral and cellular immune mechanisms, with CD4+ T cells predominating in periductal infiltrates and contributing to B-cell hyperactivity and autoantibody production [13].

While LESA is a histopathological diagnosis, SS is a clinical syndrome defined by a combination of symptoms, serological markers (anti-SSA/Ro, anti-SSB/La), and histological findings (focus score ≥ 1 per 4 mm^2^). Notably, up to 25% of SS patients may be seronegative for anti-SSA/SSB antibodies, and diagnosis in these cases relies heavily on biopsy findings and clinical evaluation [14].

Seronegative SS patients tend to have a later onset, lower prevalence of parotid gland enlargement, and distinct immunological profiles compared to seropositive patients. They exhibit higher proportions of CD4+ T helper cells, lower CD8+ T cells, and increased NK cell frequencies, suggesting a potential role for innate immunity in disease pathogenesis [15].

The risk of lymphoma, particularly MALT lymphoma, is significantly elevated in SS patients with LESA. Predictors of lymphoma development include persistent parotid swelling, purpura, splenomegaly, lymphadenopathy, high disease activity scores, cryoglobulinemia, positive rheumatoid factor, and low complement levels. The majority of lymphomas arising in SS are of B-cell origin, with a B:T cell ratio of 15:1, and MALT lymphoma accounting for over 90% of cases [16].

### 3.4. Spectrum and Progression to MALT (Extranodal Marginal Zone) Lymphoma

Chronic antigenic stimulation and persistent inflammation in LESA create a microenvironment conducive to lymphomagenesis. Somatic hypermutation and rearrangements in immunoglobulin heavy chain variable (IGHV) genes, along with activation of the NFκB pathway, contribute to neoplastic transformation. Genetic alterations such as TNFAIP3 (A20) mutations, t(11;18)(q21;q21), and trisomies 3, 7, and 18 are implicated in MALT lymphoma pathogenesis [17].

MALT lymphoma arising in the salivary glands typically follows an indolent clinical course. Unlike aggressive lymphomas, these tumors grow slowly and usually remain localized to the gland for long periods. As a result, patients most often present with symptoms caused by mass effect, such as painless parotid enlargement, facial asymmetry, or discomfort related to glandular swelling. In contrast, systemic “B symptoms”—fever, drenching night sweats, and unexplained weight loss—are uncommon in salivary gland MALT lymphoma because the disease rarely produces the high tumor burden or systemic inflammatory response seen in more aggressive lymphomas. This indolent behavior reflects the biology of MALT lymphomas, which arise from chronically stimulated marginal zone B cells within mucosa-associated lymphoid tissue and tend to remain confined to the site of origin. Consequently, the clinical presentation is dominated by localized glandular involvement, and systemic manifestations are typically absent unless the disease progresses or disseminates. Transformation to diffuse large B-cell lymphoma (DLBCL) occurs in 2–20% of cases and is associated with a worse prognosis. The 5-year overall survival for MALT lymphoma is high (89–91%), but long-term surveillance is essential due to the risk of relapse and transformation [18].

**Figure 2:**
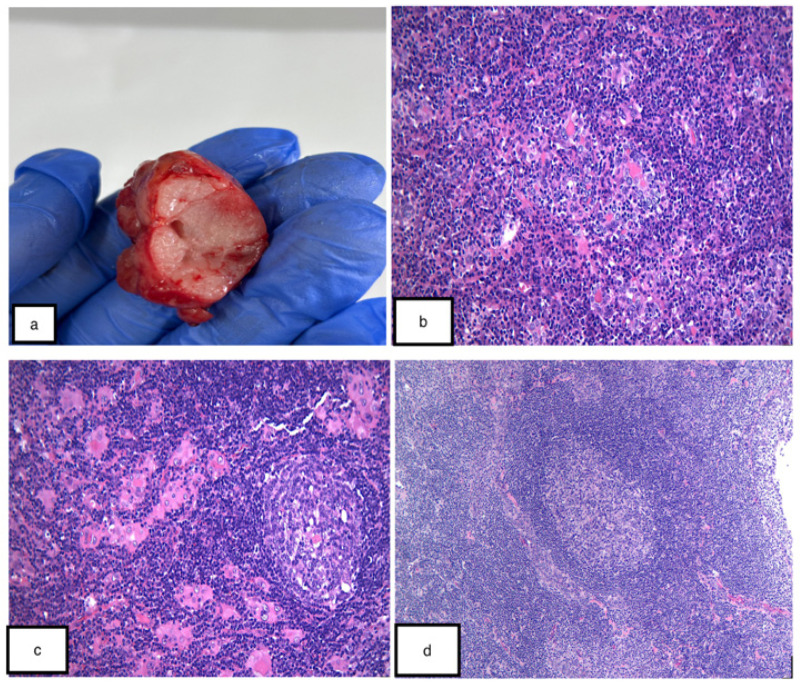
Gross aspect of the tumor (**a**). The examined slides show a proliferation of small lymphocytes with monocitoid and plasmacytoid appearance, with extensive lymphoepithelial lesion ductal hyperplasia (**b**). Numerous lymphoid follicles were also observed, with clear germinative center (**c**,**d**). HE 20×. Image from our clinical activity.

**Figure 3:**
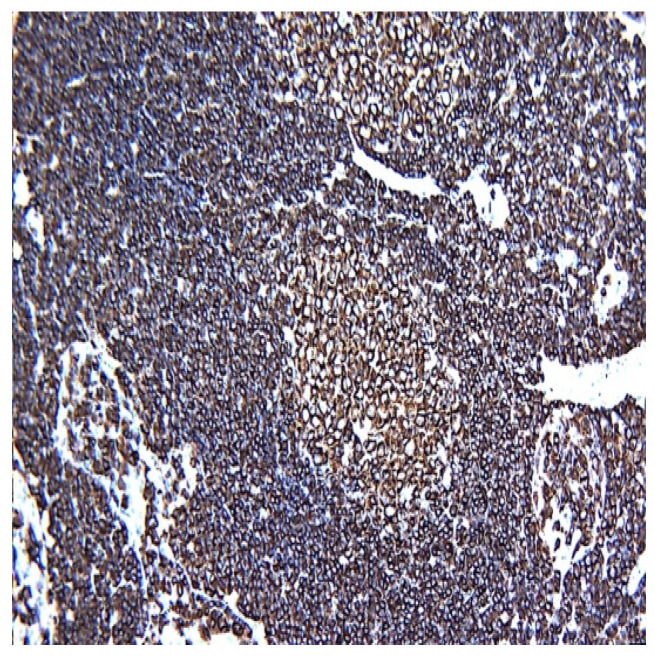
CD 20 stained intensively positive in all B cell lymphocytes in the lymphoid follicle and interfollicular areas. CD 20 immunohistochemistry, 10×. Image from our clinical activity.

**Figure 4:**
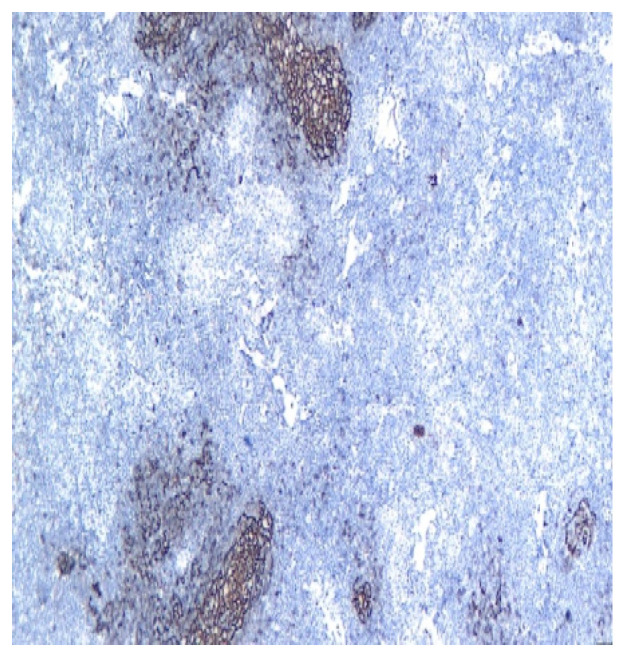
The same case presenting CD23 stain in all dendritic cells. CD 23, 10×. Image from our clinical activity.

**Figure 5:**
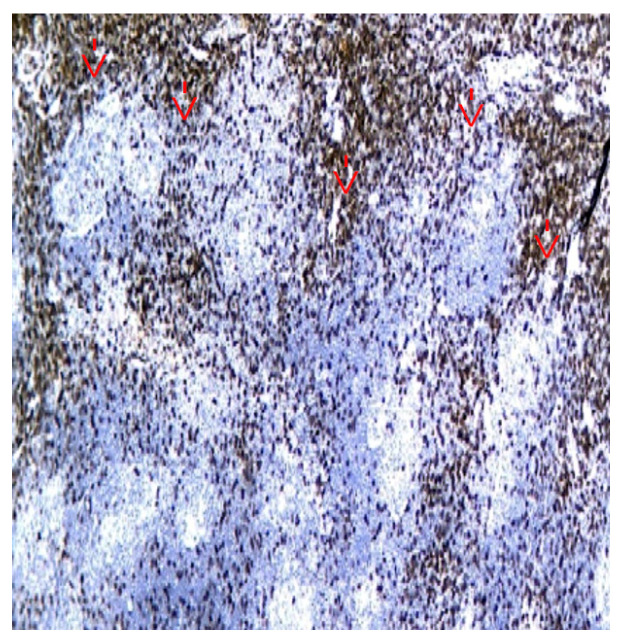
The same case presenting CD 3, highlighting (red arrows) the T cell lymphocytes from interfollicular and follicular area. CD3, 10×. Image from our clinical activity.

**Figure 6:**
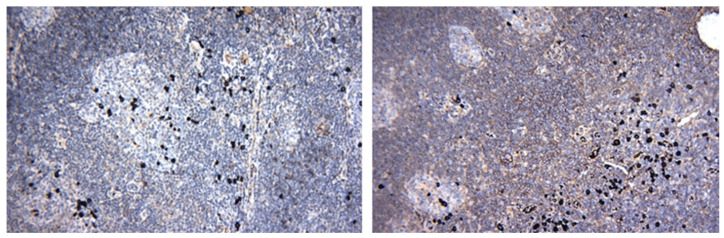
Lambda (**left figure**) and Kappa (**right figure**) immunohistochemistry markers, 10×. Kappa and lambda had normal and similar expressions, thus excluding a light chain restriction. Image from our clinical activity.

Key histological features include an atypical lymphoid infiltrate in the marginal zone, follicular colonization, and aggregates of ≥3 marginal zone cells with epithelial destruction (lymphoepithelial lesions), Figure 1. Immunophenotypically, MALT lymphoma cells are CD20+, BCL2+, CD10-, BCL6-, and may show light chain restriction, Figure 2, Figure 3, Figure 4, Figure 5 and Figure 6. Molecular evidence of clonality and characteristic chromosomal translocations support the diagnosis [19].

Figure 2, Figure 3, Figure 4, Figure 5 and Figure 6 present an example of step-by-step differential diagnosis in a LESA case.

### 3.5. Differential Diagnoses of Lymphoid-Rich Salivary Gland Lesions

The differential diagnosis of lymphoid-rich salivary gland lesions is complex due to overlapping clinical and histopathological features. Chronic sialadenitis is distinguished by fibrosis and ductal changes without lymphoepithelial lesions. IgG4-related sialadenitis presents with storiform fibrosis and abundant IgG4+ plasma cells but lacks SS-A/SS-B antibodies and is more common in men. Lymphoepithelial cysts, particularly in HIV-positive patients, are characterized by cystic architecture and dense lymphoid tissue but lack the epithelial proliferation seen in LESA [20].

Tumor-associated lymphoid proliferation may mimic LESA but is associated with a neoplastic epithelial component, as shown in Table 1.

### 3.6. Characteristics and Critical Appraisal of Included Studies

This section summarizes the main characteristics, methodological approaches, key findings, limitations, and relevance of the sources included in the scoping review. Table 2 provides a structured overview of the evidence base, allowing comparison across narrative reviews, cohort studies, case reports, consensus recommendations, and diagnostic pathology resources. This charting process supports interpretation of how each source contributes to understanding LESA diagnosis, differential diagnosis, management, surveillance, and remaining evidence gaps.

## 4. Discussions

This discussion synthesizes the charted evidence in relation to the review question and PRISMA-ScR objectives. The included literature confirms that LESA is best understood as a clinicopathological diagnosis requiring correlation of glandular symptoms, autoimmune or infectious context, imaging findings, biopsy adequacy, histopathological architecture, immunophenotype, and, in selected cases, molecular findings. The main challenge is not recognition of lymphoepithelial lesions alone, but determining whether the process is reactive, autoimmune-associated, IgG4-related, HIV-associated, tumor-associated, or lymphoma-associated. The following sections therefore organize the evidence around imaging, biopsy strategy, ancillary testing, management, surveillance, guidelines, special mimics, multidisciplinary pathways, and remaining evidence gaps.

### 4.1. Imaging Modalities

Ultrasound is often the first-line imaging modality for evaluating salivary gland masses due to its accessibility, lack of radiation, and ability to differentiate cystic from solid lesions. However, its sensitivity for detecting deep or small lesions is limited, and operator experience significantly affects diagnostic accuracy. Contrast-enhanced ultrasound (CEUS) improves differentiation between IgG4-related sialadenitis and benign masses, with higher diagnostic accuracy than conventional US [21].

CT and MRI provide superior anatomical detail and are useful for assessing the extent of disease, deep lobe involvement, and perineural invasion. MRI with diffusion-weighted imaging (DWI) can help differentiate benign from malignant lesions based on water diffusion characteristics. However, both modalities have limitations in distinguishing inflammatory from neoplastic processes, and MRI availability may be limited in some settings [22].

The role of PET in salivary gland lesions is controversial, as physiologic uptake in salivary tissue can obscure pathological findings. PET is more useful for staging high-grade or advanced malignancies [23].

### 4.2. Type of Biopsy: Diagnostic Challenges and Pitfalls

FNA is a minimally invasive, cost-effective technique widely used for the preoperative evaluation of salivary gland masses. It has high specificity (up to 97%) for detecting malignancy but variable sensitivity (57–86%), with lower accuracy in lymphoid-rich lesions due to sampling error and cytomorphologic overlap between benign and malignant processes [24]. The Milan System for Reporting Salivary Gland Cytopathology (MSRSGC) provides a standardized framework for risk stratification, but the risk of malignancy in lymphoid-rich aspirates remains significant (up to 52% in some series) [25].

Key pitfalls include overlap in cytomorphology between reactive lymphoid hyperplasia, LESA, and low-grade lymphoma; sampling bias, especially in cystic or heterogeneous lesions; and the limited ability to assess architectural features critical for lymphoma diagnosis [26].

Core needle biopsy (CNB) under ultrasound guidance provides larger tissue samples with preserved architecture, improving diagnostic accuracy for lymphoma and enabling ancillary studies (immunohistochemistry, flow cytometry, molecular testing). CNB has higher sensitivity (94%) and specificity (98%) than FNA, with a low rate of complications when performed by experienced operators. Excisional biopsy remains the gold standard for definitive diagnosis when less invasive methods are inconclusive or when malignancy is strongly suspected [27].

### 4.3. Ancillary Molecular and Flow Cytometry Testing for Clonality

Flow cytometry is the most sensitive method for establishing B-cell clonality, detecting monotypic surface immunoglobulin light chain expression (kappa or lambda) and characterizing lymphocyte subsets. It is particularly valuable in distinguishing reactive from neoplastic lymphoid proliferations in FNA or CNB specimens. However, its routine use is limited by tissue availability and the need for fresh samples [28].

Polymerase chain reaction (PCR)-based analysis of immunoglobulin heavy chain (IGH) and T-cell receptor (TCR) gene rearrangements is used to assess clonality in suspect lymphoproliferative disorders. The BIOMED-2/EuroClonality protocols provide standardized multiplex PCR assays for detecting clonal rearrangements. Monoclonality supports a diagnosis of lymphoma but is not definitive, as clonal populations may be detected in reactive conditions, especially in autoimmune diseases. Interpretation must be integrated with clinical, histological, and immunophenotypic data [29].

Immunohistochemistry (IHC) is essential for lymphoma subtyping. Recommended panels for small/low-grade B-cell lymphomas include CD3 (T-cell marker), CD20 (B-cell marker), Ki-67 (proliferation index), Bcl-2, CD10, CD5, cyclin D1, SOX11, CD23, CD21, CD138, and light chain (kappa/lambda) stains. Marginal zone lymphoma (MALT) is typically CD20+, Bcl-2+, CD10-, CD5-, CD23-, cyclin D1-, and SOX11-. Light chain restriction indicates monoclonality [30].

Distinguishing LESA from MALT lymphoma and other malignancies is a major diagnostic challenge due to overlapping features. LESA is characterized by a polyclonal lymphoid infiltrate, preserved lobular architecture, lymphoepithelial lesions with bland epithelial cells, absence of cytologic atypia, and lack of destructive growth, whereas MALT lymphoma shows a monoclonal B-cell infiltrate, follicular colonization, aggregates of ≥3 marginal zone cells with epithelial destruction, light chain restriction, and molecular or cytogenetic aberrations such as t(11;18). Other malignancies are more likely to demonstrate cytologic atypia, loss of lobular architecture, extension beyond the gland capsule, and a high proliferation index. The presence of monoclonal B-cell populations or light chain restriction in the absence of overt morphological evidence of lymphoma warrants close follow-up rather than immediate diagnosis of malignancy [31].

Serological testing is integral to the diagnosis and management of LESA and associated conditions. Key biomarkers include anti-SSA/Ro and anti-SSB/La antibodies, which are highly specific for Sjögren’s disease and are present in up to 75% of cases but absent in a significant minority with seronegative disease; rheumatoid factor (RF) and antinuclear antibodies (ANA), which are commonly positive in Sjögren’s disease but are less specific; complement levels (C3 and C4), whose reduction is associated with an increased risk of lymphoma transformation; cryoglobulins, whose presence indicates higher disease activity and lymphoma risk; serum IgG4, which is elevated in IgG4-related sialadenitis; and β2 microglobulin and monoclonal gammopathy, which are associated with advanced disease and poor prognosis in MALT lymphoma [32].

### 4.4. Management Strategy: Surveillance Protocols and Follow-Up

Given the indolent nature of LESA and the risk of lymphoma transformation, regular surveillance is essential and typically includes clinical examination and symptom assessment every 3–6 months for the first 2 years, followed by annual review; imaging with ultrasound or MRI as indicated for new or changing masses; repeat biopsy or core needle biopsy when malignant transformation is suspected, such as in cases of rapid gland enlargement or new systemic symptoms; and laboratory monitoring of serological markers and immunoglobulin levels, as shown in Table 3.

Long-term follow-up (10–20 years) is recommended for patients with a history of LESA or salivary gland lymphoma, given the risk of late recurrence or transformation.

Watchful waiting is appropriate for indolent, asymptomatic LESA and selected cases of early-stage MALT lymphoma, but it requires vigilant follow-up because of the ongoing risk of progression. Corticosteroids are effective for acute glandular swelling and pain, and short courses (e.g., prednisolone 0.5–1 mg/kg/day for 5–7 days) are preferred to minimize adverse effects; intraductal steroid irrigation via sialendoscopy is also a promising approach for symptom relief in SS and non-Sjögren’s sicca disease. Immunosuppressants and biologics are used in refractory or systemic disease, with rituximab (anti-CD20) and belimumab (anti-BAFF) showing efficacy in reducing disease activity and parotid swelling in SS. Sialendoscopy and ductal therapies are minimally invasive interventions for obstructive symptoms, strictures, and mucous plugs, and sialendoscopy with saline or corticosteroid irrigation improves salivary flow and reduces xerostomia, with high patient tolerability and low complication rates; early intervention yields better outcomes. Surgery is indicated for confirmed or strongly suspected malignancy, refractory symptoms, or diagnostic uncertainty, and superficial or total parotidectomy is performed with meticulous facial nerve preservation, although surgery is less favored for benign LESA unless necessary. Radiation and chemotherapy are reserved for lymphoma management, with involved-field radiation being effective for localized MALT lymphoma and systemic chemotherapy used for advanced or transformed diseases.

### 4.5. Existing Guidelines, Consensus Statements, and Identified Gaps

Current Guidelines like the 2019 EULAR recommendations emphasize multidisciplinary care, individualized treatment based on disease activity and organ involvement, and a stepwise therapeutic approach ranging from topical to systemic and biologic therapies. The 2021 ASCO guidelines provide evidence-based recommendations for the diagnosis and management of salivary gland malignancies, including imaging, biopsy, and surgical procedures. In addition, the Milan System for Reporting Salivary Gland Cytopathology standardizes fine-needle aspiration reporting and risk stratification [33,34,38].

Identified Gaps include the lack of standardized surveillance protocols for LESA and MALT lymphoma, limited prospective data on the efficacy of immunosuppressants and biologics in LESA and Sjögren’s disease, and uncertainty regarding the optimal use of sialendoscopy and intraductal therapies. Additional gaps include the need for validated histopathological criteria for parotid gland biopsies in SS and LESA, insufficient evidence to guide the management of seronegative SS and atypical presentations, underutilization of molecular diagnostics and flow cytometry in routine practice, and limited guidance on special contexts such as HIV-associated parotid disease and IgG4-related sialadenitis.

LESA and SS are rare but clinically significant disorders due to their association with lymphoma. The risk of lymphoma transformation is highest in patients with persistent glandular swelling, high disease activity, cryoglobulinemia, low complement levels, and positive rheumatoid factor. The cumulative incidence of lymphoma in SS is 2–5%, with MALT lymphoma being the predominant subtype. Prognosis for LESA and early-stage MALT lymphoma is generally favorable, with high overall survival rates. However, transformation to high-grade lymphoma or DLBCL portends a worse outcome. Long-term surveillance is essential for early detection and management of malignant transformation [35].

### 4.6. Special Contexts and Mimics: HIV-Associated Parotid Disease and IgG4-Related Sialadenitis

Benign lymphoepithelial cysts (BLECs) are a hallmark of HIV-associated parotid disease, presenting as painless, bilateral parotid swelling with cervical lymphadenopathy. Histologically, BLECs consist of cystic spaces lined by stratified epithelium and dense lymphoid tissue. Diagnosis relies on clinical, serological, and imaging findings, with biopsy reserved for atypical or suspicious cases. Management options include antiretroviral therapy, sclerotherapy, and surgery, with the latter reserved for refractory or suspicious lesions [4,10].

IgG4-related sialadenitis is a systemic fibroinflammatory disorder characterized by glandular enlargement, dense lymphoplasmacytic infiltrate, storiform fibrosis, and elevated serum IgG4 levels. It predominantly affects the submandibular gland and is more common in men. Differentiation from SS and LESA is based on histological, serological, and imaging criteria. Corticosteroids and rituximab are effective treatments [3,21,23].

### 4.7. LESA Association and Overlap with IgG4-Related Disease

Lymphoepithelial sialadenitis (LESA) has been reported to overlap histologically with IgG4-related sialadenitis, creating important diagnostic challenges. Both conditions share several features, including dense lymphoplasmacytic infiltration, ductal epithelial alterations, and progressive glandular destruction. These similarities can make routine histopathological examination insufficient to distinguish between the two entities. However, IgG4-related disease exhibits distinctive markers that are absent in classic LESA. These include storiform fibrosis, obliterative phlebitis, and a significant increase in IgG4-positive plasma cells, often quantified through immunohistochemistry as an elevated IgG4/IgG ratio. The presence of these features provides a critical basis for differentiation. Clinically, the overlap is significant because IgG4-related disease represents a systemic fibroinflammatory condition with potential multi-organ involvement, whereas LESA is most often associated with Sjögren disease and confined to salivary glands. Misclassification can therefore lead to inappropriate management strategies, underscoring the importance of integrating histological findings with immunohistochemical staining and clinical context. In summary, while LESA and IgG4-related sialadenitis share overlapping morphologic features, careful attention to distinguishing markers and clinicopathologic correlation is essential to avoid diagnostic confusion and to guide appropriate patient care [36].

### 4.8. Multidisciplinary Care Models, Referral Pathways, and Quality Metrics

Optimal management of LESA and related disorders requires a multidisciplinary approach involving rheumatologists, otolaryngologists, pathologists, radiologists, and, when necessary, hematologists and oncologists. Referral to centers of expertise is recommended for complex cases, suspected malignancy, or refractory disease. Shared decision-making and individualized care plans are essential for improving patient outcomes and quality of life, as shown in Table 4.

### 4.9. Clinical Translation Value

The clinical translation value of this scoping review lies in its synthesis of fragmented evidence into a practical framework for approaching lymphoepithelial sialadenitis in daily clinical care. By mapping clinical presentation, imaging findings, biopsy strategies, histopathological criteria, immunophenotypic markers, molecular considerations, management options, and surveillance needs, the review supports a more structured pathway from initial glandular swelling to final clinicopathological interpretation. This is particularly relevant because LESA is not defined by a single diagnostic test, and misclassification may lead either to overtreatment of benign inflammatory disease or delayed recognition of salivary gland MALT lymphoma.

For clinicians, the review highlights actionable warning signs that should prompt escalation of diagnostic evaluation, including persistent or asymmetric parotid enlargement, rapid growth, nodular gland changes, lymphadenopathy, systemic symptoms, cryoglobulinemia, low complement levels, monoclonal gammopathy, light-chain restriction, or molecular evidence of clonality interpreted in the appropriate morphological context. These findings can help stratify patients into lower-risk cases suitable for observation and symptomatic care versus higher-risk cases requiring core biopsy, ancillary testing, hematology input, or closer longitudinal follow-up.

For pathologists and radiologists, the review translates the evidence into diagnostic checkpoints that may reduce interpretive uncertainty. Preservation of lobular architecture, polytypic plasma cells, and absence of destructive atypical B-cell expansion support reactive or autoimmune-associated LESA, whereas architectural effacement, follicular colonization, light-chain restriction, and compatible molecular alterations increase suspicion for MALT lymphoma. Similarly, imaging should be interpreted as part of a multidisciplinary assessment rather than as an isolated discriminator, because inflammatory, autoimmune, cystic, and neoplastic lesions may share overlapping radiological patterns.

From a health-system perspective, this review supports the development of local referral algorithms and multidisciplinary pathways involving otolaryngology, rheumatology, pathology, radiology, hematology, and oncology. Such pathways could standardize when to request serology, ultrasound or MRI, fine-needle aspiration, core needle biopsy, immunohistochemistry, flow cytometry, clonality testing, or repeat biopsy. They may also improve communication in pathology reports by encouraging explicit statements on diagnostic certainty, lymphoma risk, need for clinicopathological correlation, and suggested follow-up when findings are indeterminate.

Finally, the review has translational value for future research because it identifies the evidence needed to move LESA care from descriptive interpretation toward validated clinical decision tools. Priority areas include prospective multicenter cohorts, standardized terminology, harmonized reporting of parotid and minor salivary gland biopsies, validation of molecular and flow cytometric approaches in salivary gland specimens, and consensus surveillance protocols for patients at increased risk of lymphomatous transformation. In this way, the current scoping review provides not only a map of existing evidence but also a bridge between pathology-based diagnosis and clinically usable risk-adapted care.

## 5. Limitations

This scoping review has several limitations. First, the search strategy was intentionally broad and primarily based on Google Scholar, which increased sensitivity but may have reduced reproducibility compared with searches conducted across multiple bibliographic databases such as PubMed/MEDLINE, Embase, Scopus, or Web of Science. Second, although PRISMA-ScR reporting was followed, no formal critical appraisal was performed, consistent with the mapping purpose of the review; consequently, the strength of individual evidence sources was not graded. Third, the included literature is heterogeneous, with variation in terminology, diagnostic thresholds, biopsy practices, imaging protocols, immunohistochemical panels, molecular testing, and outcome reporting. Fourth, much of the evidence consists of retrospective studies, case reports, narrative reviews, and small series, limiting generalizability. Fifth, overlap among LESA, Sjögren’s disease, MALT lymphoma, IgG4-related sialadenitis, HIV-associated lesions, chronic sialadenitis, and tumor-associated lymphoid proliferations introduces a risk of misclassification in the primary literature, particularly when older diagnostic criteria or limited ancillary testing were used. Finally, several management and surveillance recommendations are extrapolated from the Sjögren’s disease or salivary gland lymphoma literature rather than from LESA-specific prospective cohorts. These limitations reinforce the need for standardized terminology, reproducible search strategies, multicenter registries, validated diagnostic criteria, and consensus surveillance pathways.

Although the initiating trigger of Sjögren disease remains unknown, a similar uncertainty applies to lymphoepithelial sialadenitis (LESA). Authoritative pathology sources report that the etiology of LESA, particularly when occurring independently of SjD, has not been determined. LESA is characterized histologically by lymphocytic infiltration and proliferation of ductal epithelial remnants, but no specific initiating mechanism has been identified [39].

The current literature does not provide a single study directly demonstrating that somatic hypermutation (SHM) and immunoglobulin heavy chain variable (IGHV) rearrangements initiate lymphoepithelial sialadenitis (LESA). Instead, the connection is inferred from two complementary lines of evidence. Pathology sources describe LESA as a chronic autoimmune lesion of the salivary glands and recognize it as a precursor to mucosa-associated lymphoid tissue (MALT) lymphoma. In parallel, immunogenetic studies of B-cell malignancies show that SHM and IGHV rearrangements, while essential for antibody diversification, introduce DNA breaks and repair errors that can lead to oncogenic mutations and chromosomal translocations. When these processes occur in the setting of chronic B-cell activation, as seen in LESA and Sjögren disease, the risk of clonal expansion and malignant transformation increases. Thus, the proposed link between SHM/IGHV activity and LESA is best understood as a pathogenetic perspective: LESA provides the inflammatory microenvironment, while SHM and IGHV rearrangements supply the mutational substrate. Together, they explain why LESA is not only a diagnostic hallmark of Sjögren disease but also a lesion with malignant potential, even though the mechanistic connection needs further studies [40].

Although flow cytometry (FCM) is a well-established tool for detecting B-cell clonality in hematopathology, particularly through demonstration of monotypic κ or λ light-chain expression, its application in lymphoepithelial sialadenitis (LESA) has not been documented in the published literature. Diagnostic evaluation of LESA continues to rely on histopathology, immunohistochemistry, and molecular assays such as PCR for immunoglobulin heavy chain (IGH) gene rearrangements to confirm clonality. References describing LESA emphasize its role as a precursor lesion for salivary gland mucosa-associated lymphoid tissue (MALT) lymphoma but do not report routine use of FCM in this setting. Thus, discussion of FCM in LESA should be a perspective extrapolated from its established role in lymph node and bone marrow specimens, rather than as a validated diagnostic practice. This distinction is important to avoid overstating current evidence: while FCM could theoretically provide useful information in salivary gland aspirates by identifying clonal B-cell populations, its role in LESA remains conceptual until supported by dedicated studies [41].

## 6. Conclusions

This scoping review maps the available evidence on LESA and highlights the continuing difficulty of distinguishing benign autoimmune or inflammatory lymphoepithelial lesions from extranodal marginal zone B-cell lymphoma. Current evidence does not support a single definitive diagnostic criterion; instead, diagnosis requires integrated assessment of clinical presentation, serology, imaging, histopathology, immunohistochemistry, and, when available, ancillary tests such as clonality assays, fluorescence in situ hybridization, or next-generation sequencing. Molecular and flow cytometric approaches remain promising but require further validation in salivary gland specimens before they can be considered routine LESA-specific tools. Management should be individualized, ranging from observation and symptomatic treatment for stable disease to targeted intervention when systemic autoimmune disease, obstructive symptoms, diagnostic uncertainty, or lymphoma is suspected. A multidisciplinary approach involving otolaryngology, pathology, rheumatology, radiology, hematology, and oncology remains essential. Future research should prioritize standardized diagnostic criteria, harmonized reporting of biopsy and molecular findings, prospective follow-up cohorts, and consensus surveillance protocols for patients at risk of lymphomatous transformation.

## Figures and Tables

**Table 1:** Differential Diagnoses of Lymphoid-Rich Salivary Gland Lesions.

Condition	Key Features	Distinguishing Criteria
LESA (Lymphoepithelial Sialadenitis) [1,11,14,18]	Chronic lymphoplasmacytic infiltrate, acinar atrophy, lymphoepithelial lesions, preserved lobular architecture	Polyclonal infiltrate, absence of overt cytologic atypia
MALT Lymphoma [9,11,18]	Atypical lymphoid infiltrate, follicular colonization, lymphoepithelial lesions, light chain restriction	Monoclonality, molecular/cytogenetic aberrations
Chronic Sialadenitis [13,18]	Fibrosis, acinar atrophy, ductal dilation, absence of lymphoepithelial lesions	Associated with sialolithiasis, less intense inflammation
IgG4-Related Sialadenitis [3]	Dense lymphoplasmacytic infiltrate, storiform fibrosis, obliterative phlebitis, IgG4+ plasma cells	IgG4/IgG ratio > 40%, absence of SS-A/SS-B antibodies
Lymphoepithelial Cyst [4,10]	Well-defined cystic lesion, lymphoid stroma, absence of lymphoepithelial lesions	Associated with HIV, cystic architecture
Lymphadenoma [19]	Well-circumscribed, uniform epithelial components within lymphoid stroma	Absence of lymphoepithelial lesions
Tumor-Associated Lymphoid Proliferation (TALP) [5]	Salivary gland tumor with dense lymphoid infiltrate	Lymphoid infiltrate surrounds, not permeates, tumor
Lymphocytic Interstitial Pneumonia [2]	Diffuse interstitial infiltrate, polytypic lymphocytes, plasma cells	Associated with autoimmune disease, pulmonary involvement
Sarcoidosis [20]	Non-caseating granulomas, minimal lymphoid infiltrate	Clinical and laboratory evidence of systemic sarcoidosis

**Table 2:** Tabulated Evidence from Included Studies.

Author(s) & Year	Study Objective & Design	Methodology	Key Findings	Limitations	Relevance & Critique
**Zabotti et al., 2023** [1]	Review of salivary gland pathology in pSS, including LESA.	Narrative review of histology, biopsy techniques, imaging.	Defines LESA within pSS gland pathology; contrasts labial vs. parotid biopsy.	Review-based; heterogeneous sources.	Strong modern reference for LESA morphology and diagnostic workflow.
**Alunno et al., 2018** [2]	Review of lymphoma and lymphomagenesis in pSS.	Narrative synthesis of clinical and molecular data.	Links chronic sialadenitis/LESA to MALT lymphoma development.	No unified risk model.	Core reference for LESA as a precursor environment for lymphoma.
**Valim et al., 2022** [3]	Evaluate parotid swelling in pSS.	Clinical cohort; activity indices, differential diagnoses.	Shows swelling may reflect activity or sialadenosis, not always LESA/lymphoma.	Limited histologic detail.	Important for clinical triage of LESA vs. benign swelling.
**Kato et al., 2019** [4]	Imaging of non-neoplastic parotid cysts.	Retrospective CT/MRI review.	Defines imaging patterns of benign cysts mimicking LESA.	Not pSS-specific.	Useful for radiologic differential diagnosis in LESA workup.
**Thompson & Whaley, 2021** [5]	Review of lymphoepithelial carcinoma.	Clinicopathologic review.	Distinguishes carcinoma from LESA/MALT patterns.	Rare tumor; case-based.	Helps avoid misdiagnosing carcinoma as LESA.
**Kalhor et al., 2017** [6]	Salivary-type tumors of thymus.	Case series + literature review.	Shows lymphoepithelial-like patterns outside salivary glands.	Very rare; peripheral.	Conceptual relevance for lymphoepithelial morphology.
**Nocturne & Mariette, 2015** [7]	Update on pSS-associated lymphomas.	Narrative review.	Summarizes risk factors (parotid swelling, cryoglobulins, low C4).	Heterogeneous evidence.	Key link between LESA microenvironment and lymphoma.
**Wenig, 2015** [8]	Review of lymphoepithelial-like carcinomas.	Diagnostic pathology review.	EBV-related and unrelated lymphoepithelial carcinomas described.	Not salivary-specific.	Helps differentiate carcinoma from LESA.
**Dong et al., 2025** [9]	Review of salivary MALT lymphoma.	Narrative review.	Emphasizes autoimmune sialadenitis/LESA as precursor.	Regional practice differences.	Reinforces LESA–MALT continuum.
**Piyasatukit et al., 2015** [10]	HIV-associated lymphoepithelial cyst case.	Case report.	Demonstrates LESA-like lesions in HIV.	Single case.	Highlights non-pSS causes of LESA-like pathology.
**Schreuder et al., 2017** [11]	Review of extranodal MZL pathogenesis.	Molecular + clinical synthesis.	Chronic inflammation drives MZL; salivary glands included.	Broad scope.	Mechanistic basis for LESA → MALT progression.
**Papageorgiou et al., 2015** [12]	Predictive factors for lymphoma in pSS.	Observational cohort.	Identifies gland enlargement, cryoglobulins, low C4 as predictors.	Referral bias.	Direct clinical relevance for LESA risk stratification.
**Jogai, 2021** [13]	Review of lymphoreticular pathology in head/neck.	Narrative review.	Summarizes LESA, MALT, lymphomas.	Limited depth.	Good diagnostic overview for LESA.
**Barone et al., 2016** [14]	Histology of Sjögren’s syndrome.	Book chapter review.	Defines focus score, LESA-like epithelial–lymphoid interactions.	Not primary research.	Foundational histologic reference for LESA.
**Asam et al., 2021** [15]	Role of stroma/epithelium in pSS.	Translational review.	Shows epithelial/stromal drivers of ectopic lymphoid structures.	Mechanistic focus.	Deepens understanding of LESA microenvironment.
**Emfietzoglou et al., 2022** [16]	EBV-negative lymphoepithelial SCC case.	Case report + review.	Lymphoepithelial morphology can represent carcinoma.	Single case.	Reinforces need to distinguish carcinoma from LESA.
**Stergiou et al., 2020** [17]	Review of lymphomagenesis mechanisms in pSS.	Immunologic + genetic synthesis.	Highlights B-cell activation, ectopic GCs, chronic inflammation.	No quantitative risk model.	Mechanistic relevance for LESA → lymphoma.
**Thakral et al., 2015** [18]	Update on extranodal hematopoietic neoplasms.	Diagnostic review.	Differentiates lymphoma from reactive lymphoid lesions.	Broad scope.	Useful for distinguishing MALT from LESA.
**El Hussein & Khader, 2019** [19]	Cytology of oncocytic salivary lesions.	Case-based cytology review.	Oncocytic lesions may coexist with lymphoid infiltrates.	Limited LESA focus.	Peripheral but relevant for FNA differential.
**Hernandez-Prera, 2022** [20]	WHO 5th edition update.	Classification summary.	Updates lymphoma categories and criteria.	Not salivary-specific.	Supports standardized lymphoma classification in LESA contexts.
**Trevisani et al., 2019** [21]	Diagnostic recommendations for pSS (glandular).	Systematic review + consensus.	Clarifies biopsy indications and interpretation.	Regional nuances.	Important for biopsy use in suspected LESA.
**Giannouli & Voulgarelis, 2014** [22]	Predicting lymphoma progression in pSS.	Expert review.	Summarizes biomarkers and clinical predictors.	No formal model.	Direct relevance for LESA patient monitoring.
**Parisis et al., 2020** [23]	Review of pSS as autoimmune exocrinopathy.	Broad narrative review.	Describes glandular inflammation and lymphoid structures.	Broad scope.	Contextualizes LESA within exocrine pathology.
**Barone et al., 2015** [24]	Value of salivary gland biopsy in pSS.	Narrative review.	Highlights diagnostic/prognostic value of LESA and focus score.	Expert opinion.	Practical guidance for interpreting LESA biopsies.
**Gomez et al., 2024** [25]	Milan System experience in salivary cytology.	Retrospective cytology study.	Standardized reporting improves diagnostic accuracy.	Cancer-center bias.	Useful for FNA of LESA-like lesions.
**Sundling & Kurtycz, 2019** [26]	Review of cytopathology terminology systems.	Narrative review.	Benefits of standardized reporting.	Not salivary-specific.	Supports structured reporting of LESA cytology.
**Miki et al., 2021** [27]	Summary of international cytology reporting systems.	Comparative review.	Defines categories and risk of malignancy.	High-level.	Helps integrate LESA into Milan/Bethesda frameworks.
**Bombardieri & Pitzalis, 2012** [28]	Review of ectopic lymphoid neogenesis in pSS.	Mechanistic review.	Chemokines drive ectopic GCs → LESA → lymphoma risk.	Pre-omics era.	Mechanistic foundation for LESA biology.
**Stergiou et al., 2022** [29]	Review of lymphoma biomarkers in pSS.	Expert synthesis.	Identifies candidate biomarkers for early detection.	Limited validation.	Supports precision medicine in LESA patients.
**Rizzo et al., 2020** [30]	Innate immune cells in pSS.	Translational review.	Innate immunity contributes to chronic gland inflammation.	Limited clinical correlation.	Adds depth to inflammatory drivers of LESA.
**McHugh, 2017** [31]	Salivary gland pathology overview.	Textbook chapter.	Covers inflammatory and lymphoid lesions including LESA.	Not primary data.	Practical histologic reference.
**Goules & Tzioufas, 2019** [32]	Predictive biomarkers for lymphoma in pSS.	Narrative review.	Proposes biomarker-based risk stratification.	Evidence evolving.	Relevant for LESA risk prediction.
**Ramos-Casals et al., 2020 (EULAR)** [33]	Management recommendations for pSS.	Systematic review + consensus.	Treatment algorithms for glandular/systemic disease.	Therapy-focused.	Indirect relevance for managing active LESA-like inflammation.
**Geiger et al., 2021 (ASCO)** [34]	Guideline for salivary gland malignancy.	Systematic review + consensus.	Defines management of carcinomas and lymphomas.	Oncology-focused.	Relevant when malignancy arises in LESA background.
**Hang (PathologyOutlines)** [35]	Milan reporting system summary.	Structured online reference.	Clarifies Milan categories and ROM.	Web resource.	Practical for reporting LESA-like FNAs.
**Triantafyllou et al., 2014** [36]	Comparative salivary vs. breast pathology.	Morphologic review.	Highlights shared microstructural responses to inflammation.	Limited LESA focus.	Conceptual relevance for glandular inflammatory remodeling.
**Song et al., 2026** [37]	Immunotherapy-induced sialadenitis: Sjögren’s or new entity?	Clinical–pathologic analysis of ICI-treated patients.	Identifies LESA-like sialadenitis triggered by immunotherapy; distinct immunologic signature vs. pSS.	Small cohort; variable regimens.	Crucial for distinguishing true LESA from ICI-induced mimics.

**Table 3:** Treatment Modalities for LESA and Related Disorders.

Modality	Indication	Notes
Watchful Waiting [22,30]	Asymptomatic, indolent LESA	Close surveillance for lymphoma transformation
Corticosteroids [21,23]	Symptomatic gland swelling, acute exacerbations	Short courses preferred; avoid long-term use
Immunosuppressants [24]	Refractory or systemic disease	Cyclophosphamide, azathioprine, methotrexate, mycophenolate
Biologics (Rituximab, Belimumab) [29]	Severe, refractory SS or lymphoma	B-cell-targeted therapy; evidence for efficacy in SS
Sialendoscopy and Ductal Therapies [31]	Obstructive symptoms, ductal strictures	Minimally invasive, gland-preserving, may include steroid irrigation
Surgery (Parotidectomy) [32]	Suspicion or confirmation of malignancy, refractory symptoms	Facial nerve preservation is critical; risk of complications
Radiation Therapy [9,11]	Early-stage MALT lymphoma, unresectable disease	Involved field radiation; risk of xerostomia
Systemic Chemotherapy [7]	Advanced lymphoma, high-grade transformation	CHOP, rituximab-based regimens

**Table 4:** Diagnostic Criteria for LESA, MALT Lymphoma, and Major Mimics.

Feature	LESA [1,4,11,14]	MALT Lymphoma [37]	IgG4-Related Sialadenitis [3,21,23,31]	HIV-Associated BLEC [4,10,20,31]
Clinical Presentation	Recurrent, firm swelling; often bilateral; may have sicca symptoms	Mass lesion, indolent course; may have B symptoms	Painless, bilateral gland enlargement; mild dysfunction	Bilateral parotid swelling, cervical lymphadenopathy
Histology	Chronic lymphoplasmacytic infiltrate, acinar atrophy, lymphoepithelial lesions, preserved lobular architecture	Monoclonal B-cell infiltrate, follicular colonization, lymphoepithelial lesions, light chain restriction	Dense lymphoplasmacytic infiltrate, storiform fibrosis, obliterative phlebitis, IgG4+ plasma cells	Cystic spaces lined by stratified epithelium, dense lymphoid tissue
Immunophenotype	Polyclonal T and B cells, polytypic plasma cells	CD20+, Bcl-2+, CD10-, CD5-, CD23-, cyclin D1-, SOX11-, light chain restriction	IgG4/IgG ratio > 40%, absence of SS-A/SS-B antibodies	HIV-1 p24 antigen in follicular dendritic cells
Molecular	Polyclonal or oligoclonal IGH rearrangement	Monoclonal IGH rearrangement, t(11;18), t(14;18)	Nonspecific	Nonspecific
Serology	May have anti-SSA/SSB, RF, ANA	Monoclonal gammopathy, elevated β2 microglobulin	Elevated serum IgG4	HIV serology positive
Imaging	US: hypoechoic, poorly defined; MRI/CT: deep, ill-defined mass	Variable; may mimic benign or inflammatory lesions	US/CEUS: hypoechoic, abundant blood flow	US/CT: multiple cysts

## Data Availability

All data are available upon reasonable request from the corresponding author.

## Abbreviations

The following abbreviations are used in this manuscript:

ANA	Antinuclear antibody
ASCO	American Society of Clinical Oncology
BLEC	Benign lymphoepithelial cyst
CEUS	Contrast-enhanced ultrasound
CNB	Core needle biopsy
CT	Computed tomography
DLBCL	Diffuse large B-cell lymphoma
DWI	Diffusion-weighted imaging
EULAR	European Alliance of Associations for Rheumatology
FISH	Fluorescence in situ hybridization
FNA	Fine-needle aspiration
HE	Hematoxylin and eosin
HIV	Human immunodeficiency virus
IHC	Immunohistochemistry
IGH	Immunoglobulin heavy chain
IgG4	Immunoglobulin G4
LEL	Lymphoepithelial lesion
LESA	Lymphoepithelial sialadenitis
MALT	Mucosa-associated lymphoid tissue
MRI	Magnetic resonance imaging
MSRSGC	Milan System for Reporting Salivary Gland Cytopathology
NGS	Next-generation sequencing
PCR	Polymerase chain reaction
PET	Positron emission tomography
RF	Rheumatoid factor
SS	Sjögren’s syndrome
TALP	Tumor-associated lymphoid proliferation
TCR	T-cell receptor
US	Ultrasound
EMZBCL	Extranodal marginal zone B-cell lymphoma
